# Supplementary material for: The genome sequence of the Toadflax Brocade, Calophasia lunula (Hufnagel, 1766) (Lepidoptera: Noctuidae)
Source: Wellcome Open Res. 2026 Sep 24;11:737. [Version 1] doi: 10.12688/wellcomeopenres.27918.1 (PMC13650317; doi:10.12688/wellcomeopenres.27918.1)
Supplement: Peer review history

# Reviewer report: Reviewer response for version 1

**DOI:** 10.21956/wellcomeopenres.30718.r170620

**Published:** 2026-10-16

**Authors:** Richard A Fandino

**Affiliations:** 1 BCIRL, USDA-ARS Biological Control of Insects Research Laboratory (BCIRL), Columbia, Missouri, USA; 2 Cornell University, Ithaca, USA

This is a clean chromosome level assembly for Calophasia lunula from the Darwin Tree of Life pipeline. QV 64.6, BUSCO 99.0% with low duplication, and 99.85% in 32 chromosomes. Methods are well documented and the work is technically sound.

 My one comment is about the B1 chromosome. It’s called a supernumerary chromosome in the abstract and tables but “putative” in the text. It’s also missing from the Hi-C map in Figure 3, and in Figure 4 it only paints as M22 even though the text says it comes from chromosomes 19 and 22. A line on what supports it as a B chromosome rather than a leftover haplotig would help, such as coverage or Hi-C. If that isn’t available, “putative” should be used throughout.

 Minor: Table 4 shows “#” where “≥” should be, there are a few double periods after “et al.”, and there’s a broken Marine Biological Association link in the author information.

 The assembly is well supported, and the B1 wording is the only thing I’d want fixed.

Are the datasets clearly presented in a usable and accessible format, and the assembly and annotation available in an appropriate subject-specific repository?

Yes

Are sufficient details of the sequencing and extraction, software used, and materials provided to allow replication by others?

Yes

Are the rationale for sequencing the genome and the species significance clearly described?

Yes

Are the protocols appropriate and is the work technically sound?

Yes

Reviewer Expertise:

Molecular biology; insect functional genomics; CRISPR genome editing in non-model insects; chemosensory receptor biology

I confirm that I have read this submission and believe that I have an appropriate level of expertise to confirm that it is of an acceptable scientific standard.

# Reviewer report: Reviewer response for version 1

**DOI:** 10.21956/wellcomeopenres.30718.r170619

**Published:** 2026-10-08

**Authors:** Jurate De Prins

**Affiliations:** 1 Science Committee, Global Biodiversity Information Facilities, Brussels, Belgium

This species is common in Belgium, and it is considered Least Concern on the IUCN Red List of Flanders. Probably it might be interesting for the reader to know its host plant *Linaria vulgaris* Mill and other species of *Linaria* (Plantaginaceae).

 From a karyological point of view, it is very interesting that it contains a B1 supernumerary chromosome. Maybe it is possible to say something about certain genes attached to this chromosome or the genetic function of supernumerary chromosomes.  It is not so often the case, especially in moth karyotypes, so there is much room for exploration in the future.

Are the datasets clearly presented in a usable and accessible format, and the assembly and annotation available in an appropriate subject-specific repository?

Yes

Are sufficient details of the sequencing and extraction, software used, and materials provided to allow replication by others?

Yes

Are the rationale for sequencing the genome and the species significance clearly described?

Yes

Are the protocols appropriate and is the work technically sound?

Yes

Reviewer Expertise:

Taxonomy, Lepidoptera, Bioinformatics, Karyology

I confirm that I have read this submission and believe that I have an appropriate level of expertise to confirm that it is of an acceptable scientific standard.

# Reviewer report: Reviewer response for version 1

**Published:** 2026-09-24

**Authors:** Automated Benchmarking

**Affiliations:** 1 Wellcome Sanger Institute, Hinxton, UK

As this article is a Genome Note, Automated Benchmarking has been used to determine whether the reported assembly passes four defined checks established by the Earth BioGenome Project: the raw and assembled data are publicly available, the Earth BioGenome Project three-part metric is met, the k-mer completeness exceeds 90%, and the BUSCO score passes the stated threshold. For Calophasia lunula (ilCalLunu1), all four checks passed. Data available: pass. ENA study PRJEB79702, raw-read accessions ERR13650038, ERR13654235, and ERR14792849, and assembly accessions GCA_964276575.1 and GCA_964276645.1 are public. Earth BioGenome Project three-part metric: pass. Haplotype 1 is reported as 6.C.Q64, meeting the 6.C.Q40 reference standard. k-mer completeness: pass. The combined haplotype assemblies have 99.8% k-mer completeness, exceeding the Earth BioGenome Project target of 90%. BUSCO: pass. Haplotype 1, which is proposed as the reference assembly for this species, has 99.0% complete BUSCOs (98.3% single-copy and 0.8% duplicated). BUSCO software version: 5.5.0; lineage dataset: lepidoptera_odb10. It meets the target of greater than 90% complete and single-copy BUSCOs. This decision uses values reported in the Genome Note and public accession records.

Reviewer Expertise:

NA

I confirm that I have read this submission and believe that I have an appropriate level of expertise to confirm that it is of an acceptable scientific standard.
